# Increasing Resiliency in U.S. Air Force Personnel: A Multi-Site Trial Protocol

**DOI:** 10.1016/j.conctc.2025.101507

**Published:** 2025-06-06

**Authors:** Stephen H.A. Hernandez, Jacqueline Killian, Mark B. Parshall, Tonya Y. White, Enesha J. Hicks, Victoria Hughes, Theresa A. Bedford, Yiliang Zhu

**Affiliations:** a University of New Mexico, College of Nursing, MSC07 4380, Box 9, 1 University of New Mexico, Albuquerque, NM 87102, USA; b School of Nursing, University of Nevada, Las Vegas, Mail Stop: 3018, 4505 S. Maryland Pkwy. Las Vegas, NV 89154, USA; c 59 Medical Wing, Sciences and Technology, 1632 Nellis St., Bldg. 5406, JBSA-Lackland TX, 78236, USA; d Johns Hopkins University, School of Nursing, 5525 N. Wolfe St., Room S419, Baltimore MD 21205, USA; e 88^th^ Medical Group, Clinical Investigations, 4881 Sugar Maple Dr, Wright-Patterson AFB, Dayton, OH 45433, USA; f University of New Mexico, School of Medicine, MSC10 5550, 1 University of New Mexico, Albuquerque, NM 87102, USA

**Keywords:** Air force, Military, Resilience, Stress

## Abstract

The purpose of this study is to examine the efficacy of the Stress Management and Resilience Training (SMART) in increasing the resilience of U.S. Air Force personnel. We aim to recruit up to 500 active component Air Force personnel and provide a two-arm randomization modality to make SMART more accessible and adaptive to the personnel's schedules. Two-arm randomization will be used to assign three sites for participants to choose in-person or computer-based training (CBT) and two sites where participants are randomized into their training type (in-person or CBT). The use of two-arm randomization will enable the examination of the difference between real-world settings within the framework of causal inference, as well as, differences based upon self-selection and a randomized control trial. We propose to examine the intervention effects at 12, 24 and 36-weeks post-intervention. Initial analysis will include descriptive statistics to characterize demographic status, military grade, duty location, and military occupation. The objectives of our analyses will include testing and estimating the intervention effects by comparing pre-post intervention changes in resilience, stress, anxiety, and QoL at each follow-up. Scores will also be pooled to test for overall intervention effects over time. Intervention effectiveness will be reported by comparing mean or median effects using 95 % confidence intervals and effect size estimates. An analysis of the longitudinal trend over the study period will be conducted by simultaneously examining data from all follow-ups using mixed-effects models in which random effects will be used to characterize between and within-subject variations.

## 1 Introduction

With the increasing likelihood of future military conflicts, helping military service members to develop higher levels of psychological resilience is necessary to ensure a fit and ready force. Psychological resilience is defined as “the process of coping with or overcoming exposure to adversity or stress” (p. xiii) [1] or a “process of […] negotiating, adapting to, or managing significant sources of stress or trauma” (p. 152) [2]. Providing training to increase psychological resilience among military service members is necessary to enhance protective factors against psychological problems, such as by “hardiness, personal control, and positive coping strategies” (p. 47) [3]. The Department of Defense offers training and resources to enhance service member resilience; however, there is insufficient evidence that demonstrates the effectiveness of these programs [4,5].

The Stress Management and Resilience Training (SMART) program focuses on incorporating principles related to gratitude, mindful presence, kindness, and developing a resilient mindset [6]. SMART is provided through a structured program with a curriculum that involves three inter-related steps of increasing awareness of how the brain's normal functioning can impact individual's stress and resilience, understanding the relationship between stress and resilience with well-being, and providing relevant insights and skills which can be practiced in work and personal settings [7]. During the SMART course, students also learn about the three predispositions (deficits in focus, preoccupation with fear, and fatigue) that greatly increase stress [8]. Throughout the SMART program, participants are taught practices that have been found to improve coping, thinking, realism, behavioral control, and altruism [1,8]. These practices provide strategies to develop deeper, focused, and undistracted attention; principles to anchor thoughts; and emotional intelligence to foster deeper relationships [8].

The SMART program has shown evidence of efficacy in several studies with civilian populations [[9], [10], [11], [12], [13]]. In 2021, we completed a preliminary, randomized preventive trial involving 56 active component, United States Air Force health personnel where SMART was provided to via a 2-h training session or a self-paced, computer-based training (CBT) [14]. Participants' median resilience scores, measured by the Connor-Davidson-10 item (CD-10) scale, significantly increased by 4-, 6-, and 5-points at weeks 12, 18, and 24 post-intervention completion [14]. No statistically significant differences were found post-intervention between in-person and CBT groups in the CD-10 changes [14].

A multi-site trial is desirable to further test SMART efficacy in a real life setting with active military service members, as well as, assess for a longer-term impact on resilience and stress. In this paper we describe the design and implementation of an ongoing, multi-site randomized pragmatic trial of SMART for US Air Force personnel assigned to five Air Force bases.

## 2 Methods

### 2.1 Overview

We have designed and initiated a two-stage randomized pragmatic trial for Air Force personnel assigned to five Air Force bases. We randomly assigned three sites to a self-selection arm in which prospective participants choose in-person or CBT SMART training (the pragmatic arm) and randomly assigned two sites to a random-selection arm where participants are randomized into the in-person or CBT training type. In addition to reducing sampling bias and facilitating causal analysis, this two-stage randomization scheme will enable examination of the comparative efficacy of SMART in real-world settings and differences between self-selection and randomization of intervention modality [15].

Assessment of resilience is implemented through a pre- and post-training questionnaire, which was reviewed and approved by the Defense Health Agency (Survey Exempt Number: DHA‐1072‐EUNM). The protocol and subsequent modifications were reviewed and approved by the University of New Mexico Health Sciences Institutional Review Board (22–317). A secondary review of the protocol and all subsequent modifications was completed by the 59th Medical Wing Human Research Protections Office (FWH20230066X). The study was registered with clinicaltrials.gov (NCT05700435).

### 2.2 Participants

The military commanders at the five study sites, in Idaho, 10.13039/100017027Maryland, Nevada, Ohio, and 10.13039/100008562Texas, provided a letter of support for the study. The majority of participants are expected to be officer or enlisted personnel who had medical, nursing, or allied health training. To participate in this study, participants must be active component, U.S. Air Force personnel assigned to one of the five study site units. Participants also had to be at least 18 years of age and be able to consent to participate in the study. Basic Military Trainees are excluded from the study.

### 2.3 Screening and recruitment

The letters of support from site commanders included permission to recruit potential participants with posted flyers, newspapers, social media, and informational e-mail announcements. Recruitment materials were distributed in accordance with any limitations set by the governing institutional review boards and any site commander's instructions. Study team members were available at each site to meet with and recruit potential participants prior to enrollment in the study to provide study information. Although some study investigators were members of the active and reserve components of the Air Force, none were in the chain of command of any of the potential participants.

After a study team member ensured a potential participant met the inclusion criteria, a team member reviewed the consent document with the participant via phone, in-person, or video-teleconference and answers participant questions. Each potential participant received a copy of the consent document. The governing IRB determined the study review category was expedited, because of the minimal risks to study participants. With that determination, a waiver of signed informed consent was approved.

### 2.4 Random assignment

For the randomized assignment arm, participants were randomly assigned to the in-person or CBT modality. Due to the intervention's nature, investigator and participant blinding was not feasible. Randomization was stratified with respect to military grade (i.e. Officer or Enlisted grades) to reduce potential confounding effects. Because of the realities of military responsibilities and lifestyle (e.g. operational requirements, deployments, permanent change of station), requests for switching assignment within the randomized assignment arm were allowed and tracked to inform the future analyses.

### 2.5 Intervention

In-person instructors completed the Certified Resilience Trainer Program provided by the Global Resilience and Inner Transformation Institute [16]. The first part of the instructor training consisted of two days of in-person or online training [16]. The initial training was followed by six months of distance-learning and mentorship provided by a certified master trainer [16]. In order to assure curriculum fidelity, the Global Resilience and Inner Transformation Institute provides a standardized presentation of SMART. All study instructors met for three sessions prior to the study initiation to agree on the finalized presentation format to enhance and standardize training delivery.

The in-person SMART training was taught to participants over approximately 2 h. The training was comprised of four modules: gratitude, mindful presence, kindness, and resilient mindset [6]. If needed, the in-person training was conducted via video-teleconferencing, using the same training materials as an in-person training session. In-person sessions were limited to a maximum of 10 participants per class. The SMART CBT provides the same content as the in-person training; however, it is designed to be completed over a self-paced period of four to eight weeks [6]. Participants in the CBT group were provided a code to access the training website after they have completed the informed consent process. At the end of the in-person and CBT training, participants were provided a copy of *The Resilience Journal: A 2-Minute Commitment to Lift Your Entire Day*. This book served as a review of the practices and allowed participants to track their use of practices for improving gratitude, mindful presence, kindness, and developing a resilient mindset.

### 2.6 Evaluation and data Collection

After completion of informed consent and prior to the initiation of training, a baseline survey was administered to collect demographic information and baseline measurements with the Connor Davidson-10 Item scale (CD-10), Perceived Stress Scale (PSS), Generalized Anxiety Disorder Scale (GAD-7), and an overall quality of life (QoL). Participants received an e-mail with an individualized survey link to access the survey hosted in the Research Electronic Data Capture System (REDCap™). Follow-up surveys were sent via REDCap™ at 12, 24, and 36 weeks after completing the SMART intervention to assess the longevity of the effects of SMART on resilience and stress. Each survey was estimated to take approximately 10–15 min to complete. If the participant did not respond to the initial REDCap™ invitation for each survey, a maximum of three e-mail reminders (one reminder per week after the initial e-mail) are sent to the participant [17]. All data will be de-identified prior to the future data analysis.

### 2.7 Variables

#### 2.7.1 Outcome variables

Change in resilience scores measured by the CD-10 is the primary endpoint of the study; secondary endpoints included changes in the PSS, GAD-7, and the QoL measures. Although stress, anxiety, and QoL are not primary outcome variables in this study, a decrease in stress and anxiety and an increase in QoL have been reported in past studies by participants who completed SMART. Including the PSS, GAD-7, and QoL measure offered a more comprehensive battery of mental health, at minimal response burden, to compare with outcomes reported in previous studies.

The Connor Davidson-10 Item scale is a validated, unidimensional scale to measure resilience that is derived from the 25-item Connor Davidson Resilience Scale [18]. Each item uses a five-point numeric rating ranging from *not true at all* (0) to *true nearly all the time* (4) [18]. A total CD-10 score is calculated by summing the score of all 10 items for a total possible score of 40, with a higher score reflecting a greater level of resilience [18]. The CD-10 has a reported Cronbach's alpha of .85, which indicates good internal consistency reliability [18]. In an unpublished analysis using data collected from Air Force registered nurses and medical technicians [19], participant Connor Davidson Resilience Scale responses were reanalyzed using the items included in the CD-10, and the Cronbach's alpha was .85. Additionally, the correlation between the full Connor 10.13039/100020494Davidson Resilience Scale and the CD-10 was high (*r* = .929, *p* < .001), which supports validity of the briefer version.

The Perceived Stress Scale was developed to provide a global measure of perceived stress [20]. The PSS is a 14-item instrument, and respondents answer each item on a five-point scale ranging from *never* (0) to *very often* (4) [20]. An individual's score is calculated by reverse scoring seven items and then summing all item scores, resulting in a score range between 0 and 56 [20]. The test-retest reliability of the PSS has been reported to be .55 and .85 [20]. The PSS and Center for Epidemiological Studies Depression Scale were shown to concurrently and independently predict psychosomatic symptoms [20]. The PSS has predictive validity for social anxiety (*r* = .37, *p* < .001) and smoking cessation rates (*r* = .39, *p* < .001) [20].

The Generalized Anxiety Disorder Scale is a brief measure of anxiety. Respondents can answer each item using a four-point scale ranging from *not at all* (0) to *nearly every day* (3) [21]. A total score is calculated by summing the scores of the seven items with possible scores ranging from 0 to 21 [21]. Scores between 5 and 9 are indicative of mild anxiety, and score between 15 and 21 are indicative of severe anxiety [21]. During the scale's development, an intraclass correlation coefficient of .83 for test-retest reliability was reported [21]. Construct validity for the GAD-7 scale was demonstrated by a significant (*p* < .05 for all comparisons) positive correlations to disability days (*r* = .27), physician visits (*r* = .22), and symptom related disability (*r* = .63) [21]. The convergent validity of the scale was demonstrated by a statistically significant, positive correlations to the Beck Anxiety Inventory (*r* = .72), the anxiety subscale of the Symptom Checklist (*r* = .74), and Patient Health Questionnaire for Depression (*r* = .75) [21]. In a previous study, the SMART program demonstrated a non-statistically significant decrease in participants' reported levels of general anxiety (*Estimated Treatment Effect* = 1.74, *95 % CI* = 4.7, +1.22) [22].

A single-item linear analogue self-assessment QoL measure was used for this study to measure overall QoL using a 10-point slider ranging from *as bad as it can be* (0) to *as good as it can be* (10) [23]. The overall quality of life item has been utilized as a brief, minimal burden assessment of QoL in clinical practice and clinical trials [24]. The overall QoL mean score for an healthy individual has been reported as 8.3 (*SD* = 10.2), and a score ≤5 can indicate a clinical concern [24]. A lower overall QoL score is correlated with a worse performance status, and an increased likelihood of reporting a clinically meaningful deficit based upon a diagnosis of various types of cancers [24]. In a previous study, the SMART program demonstrated a positive effect on participants’ QoL (*Estimated Treatment Effect* = +1.2, 95 % CI = .0, 2.4, *p* = .044, *d* = .83) [13].

#### 2.7.2 Demographic characteristics

The baseline survey included demographic questions for participant age, gender, marital status, race, ethnicity, previous deployment, military grade/rank, duty location, and military job duty.

### 2.8 Data safety and security

REDCap™ is a secure and encrypted, web-based platform licensed to and managed by the University of New Mexico Health Sciences Center Clinical and Translational Science Center [25]. REDCap™ includes a suite of research tools for project management, survey administration, encrypted database storage and retrieval, and reporting [25]. E-mail contact information entered into REDCap™ by study participants will only be used for study purposes (i.e. scheduling training, providing study information, survey distribution). All personally identifiable information will be coded in REDCap™ as non-exportable data. At the end of the required storage period, all REDCap™ data files will be destroyed in a manner where the electronic data are impossible to retrieve. All paper copies of study documents (i.e. class schedules, recruitment contacts or schedules, randomization tables) will be maintained in a locked storage area located in the Site investigator's or principal investigator's offices.

## 3 Analysis of specific aims

### 3.1 Primary and ancillary analyses

In the current study, we aim to provide a two-arm modality to evaluate the efficacy of SMART with Air Force personnel. To accomplish the study aim, the following research questions will be answered.

1. To what extent does SMART increase levels of resiliency and decrease levels of stress in a sample of active component U.S. Air Force personnel?
2. Does SMART have a sustained efficacy from baseline to 12, 24 and 36-weeks after training completion in a sample of active component U.S. Air Force personnel?
3. Does SMART provided via an in-person or CBT modality demonstrate efficacy in increasing resilience and decreasing stress in active component Air Force personnel?

IBM® SPSS® Statistics (version 29 or later) and R (survey package, version 4.1.2 or later) will be used for statistical analyses. Initial analysis will include descriptive statistics, including frequencies and percentages, means and standard deviations, or medians and quartiles as appropriate, to summarize demographics and the service-related characteristics of military grade, duty location, and military occupation. Cronbach's *α* will be calculated for each multi-item scale to assess internal consistency.

The objectives of our analysis include testing and estimating the intervention effects by comparing pre-post intervention changes in resilience, stress, anxiety, and QoL at each follow-up. Both the in-person and CBT groups will be analyzed separately in addition to analyzing differences between the modalities. Scores will also be pooled to test for overall intervention effects over time. Intervention effectiveness will be reported by comparing mean or median effects using 95 % confidence intervals and effect size estimates. An analysis of the longitudinal trend over the study period will be conducted by simultaneously examining data from all follow-ups using mixed-effects models in which random effects will be used to characterize between and within-subject variations. In addition to analysis of each scale of resilience, stress, anxiety, or QOL, we will consider analysis of subscales of any specific domains of interest. To better understand factors (e.g. demographic characteristics, and military job duty) that can impact the intervention effects, our regression models on the post intervention improvements over time will incorporate these factors as covariates which may determine differential time-slopes in the models. We will also consider potential clustering effects due to study sites (i.e. Air Force Base location).

With the two-arm randomization scheme, the results of the “self-selection” group will provide an examination of the outcomes in real-world settings [26]. Comparison of the “real-world outcomes” with the results from the “randomized assignment” (intent-to-treat and per-protocol) will enable us to conduct causal inference analyses. The real difference between in-person and CBT may be used to inform a future scale-up of SMART [26].

### 3.2 Sample size estimates

Our preliminary study generated evidence of strong efficacy in both the in-person and CBT modalities of SMART. Participants reported a significant increase in resilience from baseline (*median* = 28) to 24-weeks post-intervention (*median* = 34, *p* = .003, a 21.4 % increase). Participants also reported a significant decrease in perceived stress from baseline (median = 24.5) to 24-weeks post-intervention (*median* = 16, *p* < .001, a 34.7 % decrease). Although we have observed sustained improvements in resilience and stress post-training up to 24 weeks, longer-term effects were smaller in size compared with short-term effects, and were subject to uncertainties due to attrition as follow-up continued.

Our pilot study was limited in statistical power for detecting potential determinants of variation in training effects, such as participants’ military grade and marital status. Using our pilot study results as estimates of potential training effect sizes, we conducted a power analysis based on linear mixed effects models for longitudinal training effects on resilience. The results of the simulation indicate that a sample of 425 participants is needed to achieve 80 % power for detecting differences in resilience between in-person and CBT modalities. This estimated sample size is shown to be sufficient for detecting short-term (12 weeks) versus longer term (24–36 weeks) SMART effects, for detecting potential moderation effects of age, race, marital status, military grade, or sex (all dichotomized). Therefore, we aim to recruit 500 participants from the five study sites, with 250 participants per study arm, to achieve adequate statistical power for addressing the research questions. This target sample size retains an adequate power with up to 15 % attrition.

### 3.3 Trial status

Recruiting efforts began in October 2023 and concluded in January 2025. All intervention activities have concluded. Post-intervention data will continue to be collected through April 2025. The planned data analyses will be completed by July 2025.

## 4 Discussion

This study builds upon and increases the scale of the team's pilot study focused on evaluating the efficacy of SMART in Air Force healthcare personnel. The study aims to translate our findings and demonstrate scaled-effects of SMART in increasing resilience in active component Air Force personnel. The two-stage randomization study design will allow the team to evaluate SMART in a ‘real-world’ setting that allows participants to (a) choose their training modality while (b) enabling the team to assess for causality and real differences between in-person and CBT training modalities. The study will also assess for changes in outcomes for up to 36 months to determine the need for follow-up training to sustain the initial effect(s). Additionally, undertaking a study with multiple sites with broader samples of Air Force personnel will be invaluable to informing a potential, future large-scale implementation of SMART and testing for heterogeneity intervention effects across locations.

Despite the strengths of the study, the study will have several limitations. The generalizability of the study findings to all Air Force service members or other service branch populations will be limited by the extent that the study sample is representative of the underlying recruitment population. Self-selection bias may occur, because study enrollees have self-interest in this study. In addition, differences in participants’ stress, resilience, anxiety, and quality of life scores reported during the pre-test and post-tests may be influenced by maturation or history [27]. Despite these limitations, we anticipate this study will provide evidence that SMART can enhance the psychological resilience of service members in all military branches. This study will also provide additional information, data, and evaluation of the SMART program in this unique population to inform future implementation and evaluation of resilience programs.

## CRediT authorship contribution statement

**Jacqueline Killian:** Writing – review & editing, Writing – original draft, Methodology, Investigation, Conceptualization. **Mark B. Parshall:** Writing – review & editing, Writing – original draft, Project administration, Methodology, Conceptualization. **Tonya Y. White:** Writing – review & editing, Writing – original draft, Investigation, Conceptualization. **Enesha J. Hicks:** Writing – review & editing, Writing – original draft, Investigation, Conceptualization. **Victoria Hughes:** Writing – review & editing, Writing – original draft, Investigation, Conceptualization. **Theresa A. Bedford:** Writing – review & editing, Writing – original draft, Project administration, Conceptualization. **Yiliang Zhu:** Writing – review & editing, Writing – original draft, Resources, Project administration, Methodology, Data curation, Conceptualization.

## Data statement

No data was used for the study described in the article.

## Funding support

This study is funded by a grant provided by the TriService 10.13039/100028824Nursing Research Program (Award #1189-N23-B14). The Uniformed Services University of the Health Sciences (USU), 4301 Jones Bridge Rd., A1040C, Bethesda, MD 20814–4799 is the awarding and administering office. This research was sponsored by the TriService Nursing Research Program (TSNRP), USU; however, the information or content and conclusions do not necessarily represent the official position or policy of, nor should any official endorsement be inferred on the part of, the TSNRP, USU, the Department of Defense, or the U.S. Government. The use of REDCap with this study is supported by a grant award (DHHS/NIH/NCRR #8UL1TR000041).

## Declaration of competing interest

The authors declare that they have no known competing financial interests or personal relationships that could have appeared to influence the work reported in this paper.
